# Experimental Diabetes Mellitus Exacerbates Tau Pathology in a Transgenic Mouse Model of Alzheimer's Disease

**DOI:** 10.1371/journal.pone.0007917

**Published:** 2009-11-19

**Authors:** Yazi D. Ke, Fabien Delerue, Amadeus Gladbach, Jürgen Götz, Lars M. Ittner

**Affiliations:** Alzheimer's and Parkinson's Disease Laboratory, Brain & Mind Research Institute, University of Sydney, Sydney, Australia; Massachusetts General Hospital and Harvard Medical School, United States of America

## Abstract

Diabetes mellitus (DM) is characterized by hyperglycemia caused by a lack of insulin, insulin resistance, or both. There is increasing evidence that insulin also plays a role in Alzheimer's disease (AD) as it is involved in the metabolism of β-amyloid (Aβ) and tau, two proteins that form Aβ plaques and neurofibrillary tangles (NFTs), respectively, the hallmark lesions in AD. Here, we examined the effects of experimental DM on a pre-existing tau pathology in the pR5 transgenic mouse strain that is characterized by NFTs. pR5 mice express P301L mutant human tau that is associated with dementia. Experimental DM was induced by administration of streptozotocin (STZ), which causes insulin deficiency. We determined phosphorylation of tau, using immunohistochemistry and Western blotting. Solubility of tau was determined upon extraction with sarkosyl and formic acid, and Gallyas silver staining was employed to reveal NFTs. Insulin depletion by STZ administration in six months-old non-transgenic mice causes increased tau phosphorylation, without its deposition or NFT formation. In contrast, in pR5 mice this results in massive deposition of hyperphosphorylated, insoluble tau. Furthermore, they develop a pronounced tau-histopathology, including NFTs at this early age, while the pathology in sham-treated pR5 mice is moderate. Whereas experimental DM did not result in deposition of hyperphosphorylated tau in non-transgenic mice, a predisposition to develop a tau pathology in young pR5 mice was both sufficient and necessary to exacerbate tau deposition and NFT formation. Hence, DM can accelerate onset and increase severity of disease in individuals with a predisposition to developing tau pathology.

## Introduction

Alzheimer's disease (AD) is a devastating progressive neurodegenerative disease affecting more than 15 million people worldwide [1]. It is characterized by neuronal loss associated with a progressive decline in memory and other cognitive functions, resulting in dementia. Frontotemporal lobar degeneration (FTLD) is the second most common form of dementia presenting before the age of 65 [2], [3]. FTLD is a heterogeneous group of neurodegenerative disorders that is characterized clinically by either behavioural changes, such as in personality and social conduct; and/or language abnormalities, such as aphasia [4].

In the AD brain, the β-amyloid (Aβ) peptide and the microtubule-associated protein tau undergo changes in their tertiary structures leading to self-association and deposition. Aβ is derived from the precursor protein APP and forms the major constituent of plaques; while hyperphosphorylated forms of tau are the major constituents of neurofibrillary tangles (NFTs) [5]. Tau contains multiple phosphorylation sites, that are hyperphosphorylated under pathological conditions, such as AD and FTLD [6]. Deposition of hyperphosphorylated tau in the absence of an overt Aβ pathology characterizes approximately half of all FTLD cases [2]. In Frontotemporal dementia with parkinsonism linked to chromosome 17 (FTDP-17), a subtype of FTLD, mutations were identified in the tau-encoding gene *MAPT*, such as P301L [7], [8]. We, along with others, expressed mutant human tau in transgenic mice that recapitulate major aspects of the human disease (reviewed in [9]).

Diabetes mellitus (DM) is characterized by hyperglycemia. In type 1 diabetes (insulin-dependent diabetes mellitus (IDDM)), this results from an absolute deficiency of insulin secretion [10]. In type 2 diabetes (non-insulin-dependent diabetes mellitus (NIDDM)) both insulin resistance of target organs and inadequate insulin secretion cause hyperglycemia [10]. DM is associated with pathological changes in numerous peripheral organs, such as eyes, kidneys and peripheral nerves, but it also affects the central nervous system (CNS). Specifically, learning abilities are impaired and memory deficits are evident in both types of diabetes [11]. A common feature of AD and DM is amyloid deposition in target organs; Aβ and tau in AD brains, and amylin in pancreatic islets of type 2 diabetes [12]. Amidst some controversy, there is growing evidence that having DM almost doubles the risk to develop AD, with biochemical analysis supporting a link between AD and insulin dysfunction [13]. Defective insulin signalling and altered glucose metabolism have also been found in AD [14]–[16]. Furthermore, insulin can modulate tau phosphorylation *in vitro* [17] and in non-transgenic mice, depletion of insulin results in increased tau phosphorylation, with no concomitant formation of pathological tau deposits [18], [19]. While co-morbidity is difficult to address in humans, transgenic mice offer the selective advantage to determine whether reduction in insulin levels can exacerbate a pre-existing tau pathology.

Here, we examined the effects of STZ-induced insulin depletion on the onset and progression of tau pathology in young pre-symptomatic transgenic mice (pR5) that express human tau in neurons together with the pathogenic P301L mutation. Insulin deficiency and consequently, increased glucose levels exacerbated the histopathology of pR5, with accelerated tau deposition and NFT formation.

## Results

**Figure 1:**
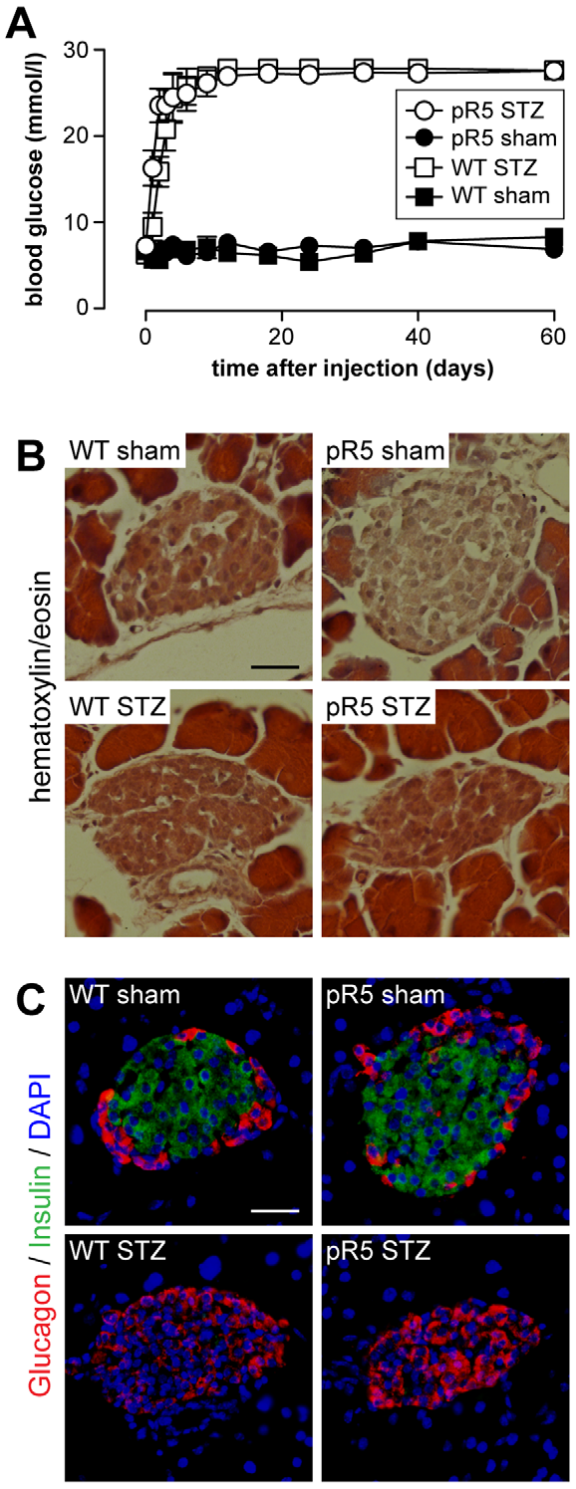
Effects of streptozotocin (STZ) administration in mice. (***A***) STZ injection in both 4 months old wild-type (WT) and pR5 mice induces chronically high blood glucose levels, while levels in sham-injected WT and pR5 control mice are normal (n = 12). Blood glucose levels remained high until mice were analyzed at 6 months of age. (***B***) Hematoxylin/eosin staining and (***C***) immunofluorescence staining with insulin- (green) and glucagon-specific (red) antibodies of pancreatic islets of Langerhans reveals comparable islet architecture in 6 months old sham-injected WT and pR5 mice. However, insulin producing β-cells are absent from both WT and pR5 islets 60 days after STZ administration, whereas glucagon secreting α-cells remained. Nuclei were counterstained with DAPI (blue). Scale bar, 25 µm.

Lack of insulin has previously been shown to result in increased phosphorylation of tau in non-transgenic mice, but failed to induce NFT formation [18], [19]. Here, we hypothesized that depletion of insulin may accelerate the tau pathology in a mouse model that is prone to develop NFTs. Therefore, we treated P301L tau transgenic (pR5) and wild-type mice as controls with STZ at 4 months of age, 2 months before NFT formation is initiated in P301L tau mice. As expected, the STZ treatment caused persistently increased blood glucose levels as pancreatic β-cells in the islets of Langerhans were destroyed resulting in depletion of systemic insulin (Figure 1A). Islets of pR5 mice were indistinguishable from those of wild-type mice, as revealed by hematoxylin/eosin staining and immunohistochemistry with antibodies to insulin and glucagon (Figure 1B,C). Accordingly, serum insulin levels of pR5 and wild-type mice were similar (0.79±0.18 ng/ml (pR5) versus 0.83±0.17 ng/ml (wild-type), n = 8). In contrast, insulin-positive β-cells were absent from islets of STZ-treated pR5 and wild-type mice, while glucagon-secreting α-cells were not affected (Figure 1C).

**Figure 2:**
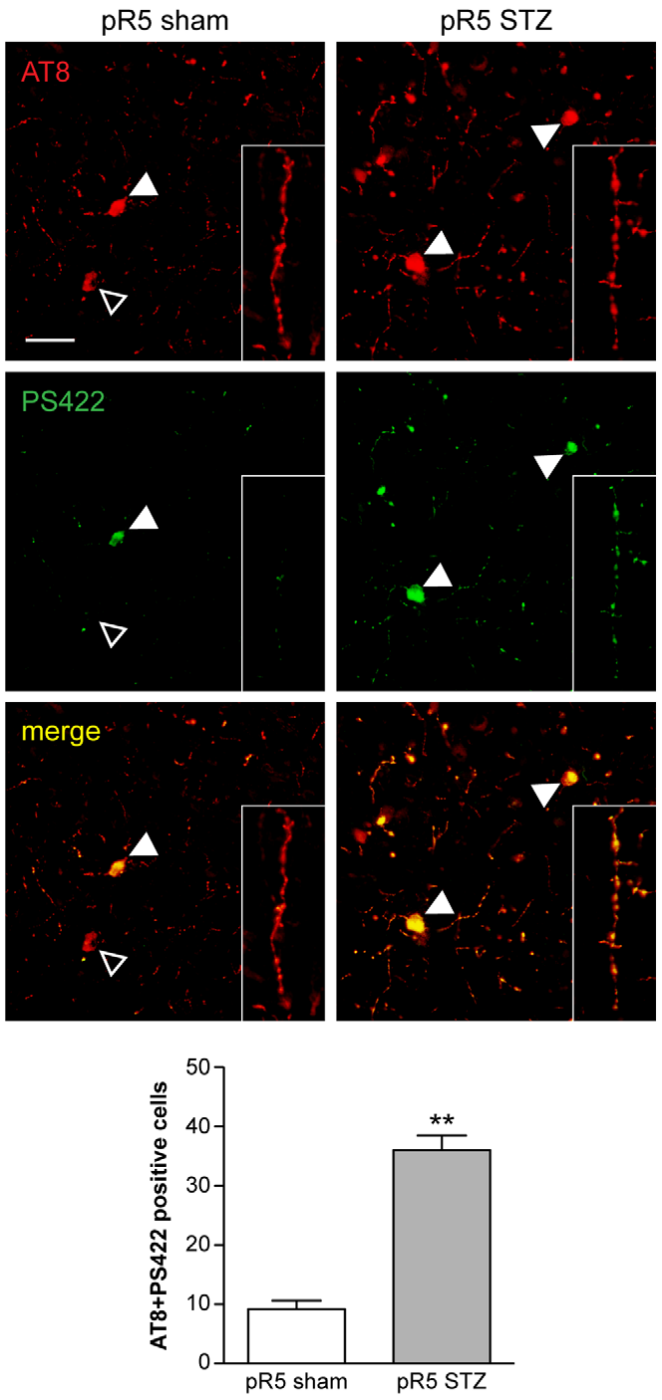
Increased tau phosphorylation in STZ-treated pR5 mice. Immunohistochemistry with phosphorylation site-specific antibodies reveals several AT8-positive neurons in the amygdala of sham-injected pR5 mice at 6 months of age. Whereas in sham-injected pR5 mice most AT8-positive cells lack PS422 staining (open arrowhead), there is a subset of neurons that also stains with PS422 (arrowhead). Similarly, dystrophic neurites were AT8- but not PS422-positive in sham-injected pR5 mice (inset). In contrast, upon STZ injection virtually all tau-containing neurons (arrowhead), and dystrophic neurites (inset) within the amygdala of pR5 mice stained with both AT8 and PS422, as revealed by overlay (yellow). Quantification of AT8 and PS422 double positive cells confirms significantly increased numbers of AT8/PS422 double positive in the amygdala of STZ-treated compared to sham-injected pR5 mice (**, P<0.001; n = 6). Scale bar, 50 µm.

To determine the effects of STZ-induced DM on the phosphorylation of tau, we performed an immunohistochemical analysis of brain sections at 6 months of age, using phosphorylation- and site-specific antibodies (Table 1). NFT formation in pR5 mice is initiated at 6 months of age [20]. Consistent with our previous findings [20], [21], sham-treated pR5 mice presented with a moderate tau pathology, including phosphorylation at the S202/T205 epitope (AT8), whereas other more pathogenic sites, such as S422 (PS422) revealed no marked phosphorylation (Figure 2). AT8-positive neurons were mainly found in the amygdala, in line with the previous finding that the tau pathology in the pR5 strain is initially restricted to the amygdala, a site where NFTs form when the mice age [22], [23]. Compared to sham-treated pR5 mice, in STZ-treated mice, more neurons in the amygdala stained positive for AT8. Importantly and different from sham treated controls, most of the AT8-positive neurons in STZ-treated pR5 mice also stained with the pS422 antibody, suggesting a more advanced pathology. Accordingly, numbers of AT8 and PS422 double positive cells were significantly increased in STZ-treated compared to sham-injected pR5 mice. Furthermore, dystrophic neurites were visualized with AT8 and pS422 in STZ-treated pR5 brains, but only with AT8 in sham-treated pR5 mice. Taken together, STZ-induced DM in pR5 mice resulted in an increased phosphorylation of tau, in particular at the pathogenic site pS422.

**Table 1:** Details of tau specific antibodies used in this study.

name	donor species	epitope/specificity	dilution	source
HT7	mouse	human tau	1∶1000 WB	Pierce
TAU5	mouse	human and murine tau	1∶1000 WB	Chemicon
AT8	mouse	Ser202/Thr205	1∶1000 WB, 1∶100 IHC	Pierce
AT270	mouse	Thr181	1∶1000 WB	Pierce
AT100	mouse	Ser212/Thr214	1∶1000 WB	Pierce
12E8	mouse	Ser262/Ser356	1∶1000 WB	Dr. Peter Seubert (Elan)
PHF-1	mouse	Ser396/Ser404	1∶1000 WB	Dr. Peter Davies
PS422	rabbit	Ser422	1∶1000 WB, 1∶200 IHC	Biosource

WB, Western blotting; IHC, immunohistochemistry.

**Figure 3:**
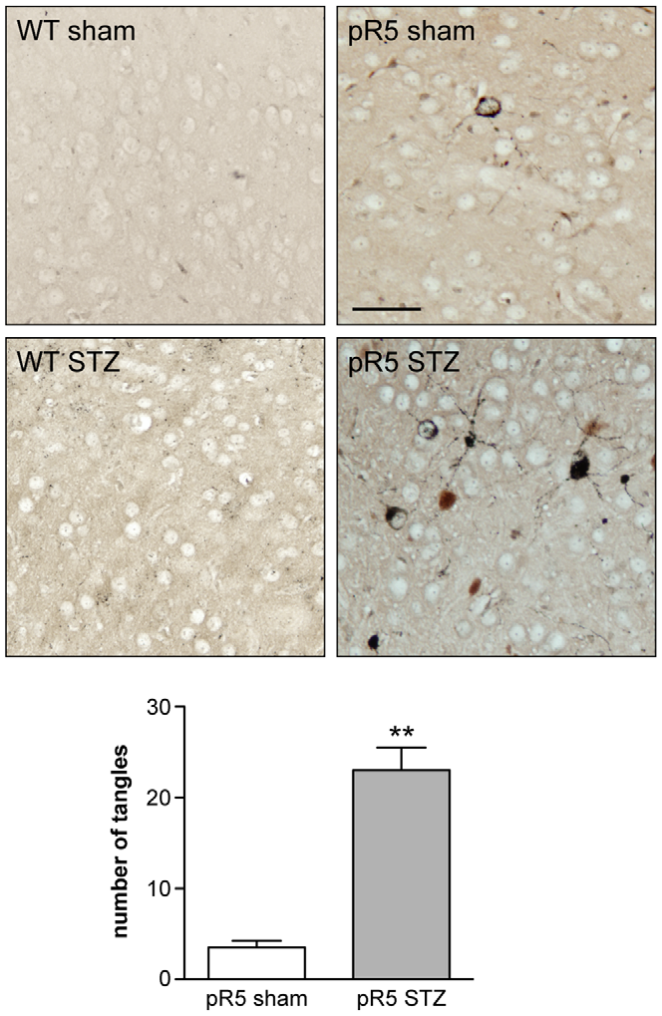
Increased numbers of NFTs in STZ-treated pR5 mice. Gallyas silver staining (black) of coronal section revealed abundant numbers of flame-shaped NFTs and dystrophic neurites in the amygdala of STZ-treated pR5 mice. In sham-injected pR5 mice, NFTs were rare. No NFTs are found in sham- and STZ-injected wild-type (WT) mice. Quantification of standardized serial sections showed 8-fold increased numbers of NFTs in pR5 mice upon STZ-treatment (**, P<0.001, n = 6). Scale bar, 50 µm.

In disease, progressive hyperphosphorylation of tau, in particular at pathologic sites, such as pS422 occurs as disease progresses, concomitant with NFT formation [6]. The abundant phosphorylation of S422 in the amygdala of pR5 mice upon STZ administration indicated that more NFTs may have been induced by the STZ treatment than are normally found at this age [20]. To address this, we used Gallyas silver impregnation that reveals filamentous tau deposits (Figure 3). As expected, NFTs were hardly ever found in sham-injected pR5 mice and completely absent in non-transgenic controls. In contrast, STZ-treated pR5 brains showed significant numbers of Gallyas-positive NFTs in the amygdala. Furthermore, dystrophic neurites were Gallyas-positive in STZ-treated, but not sham-treated pR5 brains. Taken together, Gallyas silver staining revealed advanced deposition of fibrillar tau in STZ-treated pR5 mice.

**Figure 4:**
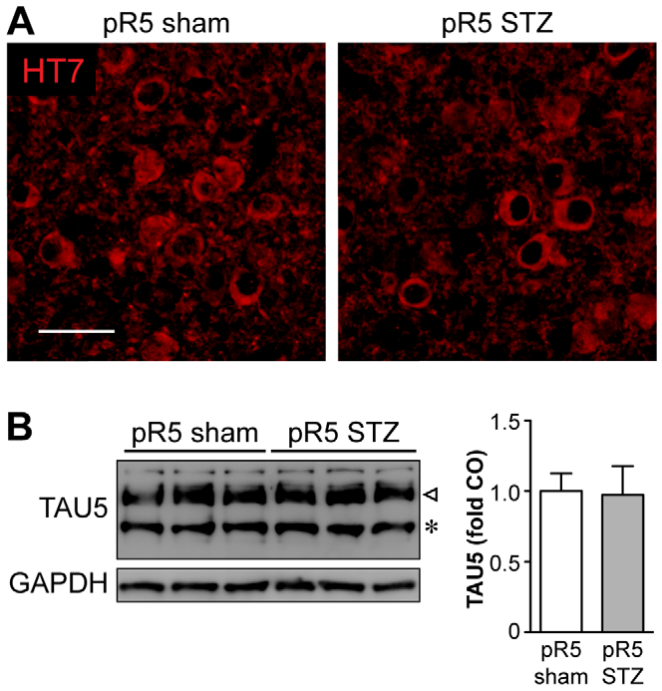
STZ administration does not affect transgene expression levels and pattern. (***A***) Immunohistochemistry using the human tau specific antibody HT7 reveals a similar expression pattern in the amygdala of STZ-treated or sham-injected pR5 mice (n = 6). Scale bar, 50 µm. (***B***) Western blotting of protein extracts from amygdala of STZ-treated or sham-injected pR5 mice (n = 6) shows comparable levels of transgenic human (triangle) and endogenous murine (asterisk) tau, as detected by TAU5. GAPDH confirms equal loading and was used to normalize TAU5 band intensities for quantification.

To rule out that STZ administration may have altered expression level or pattern of transgenic tau, we performed immunohistochemistry of brain sections and Western blotting of brain extracts, using phosphorylation-independent tau specific antibodies, HT7 and TAU5. We found that STZ treatment did not affect expression levels of transgenic tau in pR5 mice as there were no significant differences in STZ- injected compared to sham-treated pR5 mice (Figure 4).

**Figure 5:**
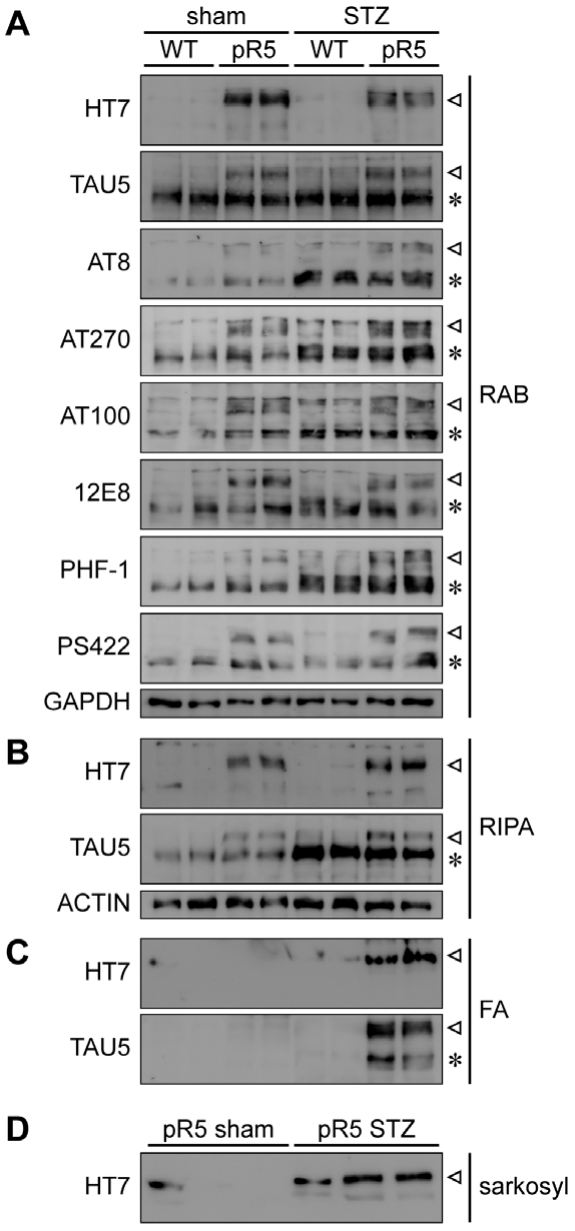
STZ-treatment induces hyperphosphorylation of tau in pR5 and non-transgenic mice, but only in pR5 mice it induced tau insolubility. (***A***) Amygdalae of STZ- or sham-injected wild-type (WT) and pR5 mice were extracted with buffers of increasing stringency and separated by SDS-PAGE for Western blotting. RAB fractions contain soluble species of both transgenic human (triangle) and endogenous murine (asterisk) tau, as shown with HT7 and TAU5, respectively. Phosphorylation site-specific tau antibodies AT8, AT270, AT100, 12E8, PHF-1 and PS422 (for details on antibodies see Table 1) reveal increased phosphorylation of transgenic and endogenous tau at multiple sites upon STZ-treatment. In sham-treated controls, tau is slightly more phosphorylated in pR5 than WT mice. GAPDH confirmed equal loading. (***B***) RIPA fractions of STZ-treated mice contain higher amounts of both transgenic and endogenous tau than in sham-treated controls. Actin confirmed equal loading. (***C***) Insoluble proteins were extracted with formic acid (FA). In STZ-treated mice, HT7 and TAU5 show insoluble transgenic and endogenous tau, whereas no tau is detectable in STZ-treated non-transgenic (WT) or sham-injected non-transgenic and pR5 mice. (***D***) Extraction of sarkosyl-insoluble tau from amygdala reveals significant amounts of sarkosyl-insoluble transgenic human tau (HT7) were isolated from STZ-treated, but not sham-injected pR5 mice.

Next, we addressed the solubility of tau, using RAB, RIPA and formic acid (FA) sequential extractions [24], [25], to determine levels of insoluble aggregates and phosphorylation of tau in brains of STZ-treated pR5 and wild-type mice. These experiments were complemented by a second protocol for the isolation of insoluble tau using sarkosyl extraction [26], [27]. Proteins from both extraction procedures were analyzed by Western blotting, using both phosphorylation-dependent and -independent antibodies (Table 1). We found that STZ administration in non-transgenic mice resulted in increased phosphorylation of endogenous murine tau at multiple sites, including T181 (AT270), AT8, T212/S214 (AT100), S262/S356 (12E8), S396/S404 (PHF-1) and, to a much lesser degree, pS422 in the RAB fraction of the amygdala homogenate (Figure 5A). Although, slightly increased amounts of tau were found in the RIPA fraction of STZ-treated non-transgenic mice compared to untreated controls, suggesting a decreased solubility of murine tau, no tau was revealed in the corresponding FA fractions of STZ-treated non-transgenic mice (Figure 5B and C). Similarly, murine tau was absent from sarkosyl-insoluble extracts of STZ treated non-transgenic brains (data not shown). Hence, insulin depletion induced tau hyperphosphorylation without formation of detectable insoluble deposits in non-transgenic mice.

In comparison, in 6 months-old sham-treated pR5 mice, both endogenous murine and transgenic human tau was phosphorylated at multiple sites including AT8, AT270, AT100, 12E8, PHF-1 and PS422 (Figure 5A). While both transgenic human and endogenous murine tau was readily detectable in these sham-treated pR5 in the RAB and RIPA fractions, as revealed by antibodies HT7 and tau-5, tau was absent from FA fractions (Figure 5C), and hardly detectable in the sarkosyl-insoluble fraction (Figure 5D). Hence, 6 months-old pR5 mice are at an age before the onset of an overt tau pathology. However upon STZ treatment, levels of RIPA-soluble transgenic and endogenous tau markedly increased, as revealed by HT7 and tau-5 (Figure 5A and B). Most importantly, HT7 and tau-5 revealed a strong signal for tau in the FA fractions of STZ-treated pR5 mice (Figure 5C). Similarly, there were significant amounts of sarkosyl-insoluble tau in these mice (Figure 5D). Despite reduced levels of RAB soluble transgenic tau (due to the shift into the RIPA- and FA-fraction), in the RAB fraction, a higher degree of phosphorylation was revealed for virtually all sites examined, suggesting that tau is increasingly hyperphosphorylated in STZ-treated pR5 mice at 6 months of age (Figure 5A).

In conclusion, increased tau hyperphosphorylation in STZ-treated pR5 mice is associated with early-onset tau deposition and NFT formation.

## Discussion

In the present study, we addressed whether metabolic changes present in DM affect tau pathology, by inducing experimental DM in a transgenic model of AD. Specifically, we showed that depletion of insulin upon STZ injection and consequently, chronically increased blood glucose levels causes hyperphosphorylation of tau, a protein associated with AD. This treatment accelerated tau pathology in pR5 mice, an established transgenic strain prone to tau deposition and NFT formation [20]. In particular, we found high levels of insoluble tau and numbers of NFTs in the amygdala of 6 months-old STZ-treated pR5 mice that are generally seen only in aged pR5 mice with an advanced pathology [20], [21]. Consistent with our previous findings [20], [21], 6 months-old sham treated pR5 mice lacked an overt tau deposition and had only very few NFTs in the amygdala, as appropriate for this early stage of disease.

Other studies addressed tau phosphorylation in response to STZ treatment, albeit in non-transgenic mice [18], [19], [28]. In these, depletion of insulin resulted in a rapid increase in tau phosphorylation three days post-injection of STZ [18] and in progressively increasing levels of phosphorylated tau up to 40 days post injection [19]. We observed similar increases in phosphorylation of endogenous tau in STZ-treated non-transgenic mice. Interestingly, this was not associated with the formation of insoluble tau aggregates or NFTs, suggesting that hyperphosphorylation of tau upon insulin depletion in itself is not sufficient to induce its deposition. Alternatively, it may be that levels of hyperphosphorylated tau were not high enough, or that there was not sufficient time allowed to induce insoluble aggregates [29]. Increased tau phosphorylation in non-transgenic mice has also been shown to be associated with decreased protein phosphatase 2A (PP2A) activity [18], [19]. In addition, as insulin depletion causes hypothermia, this may have contributed to the increased tau phosphorylation at sites such as AT8 as was observed in non-transgenic mice, since body temperatures drop after STZ injection and warming the mice prior to isolating the brain partially reverses phosphorylation [19]. Similarly, hypothermia induced by anesthesia increased tau pathology [30]. Furthermore, high glucose levels in the brain, which are significantly increased already two days after STZ administration, may contribute to the tau pathology, since rapid glucose changes are known to affect brain homeostasis. Taken together, decreased phosphatase activity, hypothermia, as well as additional metabolic alterations due to insulin deficiency and high brain glucose levels may contribute concomitantly to the increased phosphorylation of tau in STZ-injected non-transgenic mice. The relative contribution, however, remains to be elucidated.

In contrast to STZ-injected non-transgenic mice, increased hyperphosphorylation of tau in insulin-depleted pR5 mice was associated with abundant amounts of insoluble tau and 8-fold increased formation of NFTs. In fact, the tau pathology of 6 months-old STZ-treated pR5 mice was accelerated as it was comparable to that normally only found in pR5 mice above an age of one year [20], [21], or in pR5 mice intracerebrally injected with aggregated Aβ to exacerbate a pre-existing tau pathology [22]. Taken together, our findings suggest that a predisposition for tau pathology, such as in young pR5 mice, is both sufficient and necessary to induce tau insolubility and NFT formation in experimental DM. This is consistent with the fact that in a wild-type human tau transgenic mouse model that does not develop NFTs despite high levels of phosphorylated tau [25], injection of P301S mutant human tau can induce fibrilization and deposition of non-mutant tau [31]. Hence, mutant human tau seems to catalyze the formation of tau deposits *in vivo*, containing both mutant and non-mutant tau [32], [33]. While administration of STZ in rodents reliably produces hyperglycemia due to destruction of insulin secreting β-cells in pancreatic islets, other features of DM are lacking, such as immunological aspects of type 1, or insulin-resistance of type 2 diabetes [34]. Taken together, and keeping the limitations of murine disease models in mind, our data suggests that DM in humans can accelerate the progression of a pre-existing tau pathology and exacerbate disease in patients that are predisposed to develop tau pathology.

In summary, we found that defective insulin secretion accelerates onset and progression of tau pathology in tau transgenic mice that are prone to NFT formation. Given the high prevalence of DM and AD worldwide, with a significant degree of co-morbidity, our finding may have direct pathomechanistic implications in establishing a link between DM and AD.

## Materials and Methods

### Ethics Statement

All animal procedures have been approved by the University of Sydney animal ethics authorities.

### Mice

The generation of the pR5 mouse strain has been previously described [20], [35]. pR5 mice express the longest human tau isoform carrying the pathogenic P301L mutation, under control of the murine Thy1.2 promoter that drives neuronal expression of the transgene. The mice were backcrossed and maintained on the C57Bl/6J background.

### Streptozotocin Treatment

Four months old pR5 mice and wild-type littermate controls were injected i.p. with 200 mg/kg streptozotocin (STZ, Sigma, USA) dissolved in 100 mM citrate buffer, pH 4.5. Controls were injected with citrate buffer only. Mice were analyzed at 6 months of age.

### Blood Glucose and Insulin Measurements

Blood glucose levels were measured using a Freestyle Papillion Mini (Abbott, Germany) from a 3 µl drop of blood obtained by nicking the tail vein. Serum insulin levels were determined by ELISA (Shibayagi, Japan).

### Protein Extraction

Proteins of different solubility were extracted from brain in buffers of increasing stringency, using a slightly modified protocol previously described [24], [25]. Briefly, brains were harvested and the amygdala were dissected [36]. The tissues were weighed and homogenized in 10 µl/mg RAB buffer (100 mM 2-(N-morpholino) ethanesulphonic acid (MES; pH 7.0), 1 mM EDTA, 0.5 mM MgSO_4_, 750 mM NaCl, 20 mM NaF, 1 mM Na_3_VO_4_ and Complete protease inhibitors (Roche, Switzerland)) using a plastic pistil (Eppendorf, Germany), followed by passing through a 29G insulin needle (Terumo, USA). After 30 min of incubation on ice, the samples were centrifuged at 50'000×g for 20 min at 4°C. The supernatants containing RAB-soluble proteins were collected and centrifuged again. The pellets from both spins were resuspended in RAB buffer and centrifuged again. The supernatant was discarded, the pellet resuspended in 7.5 µl/mg RIPA buffer (50 mM Tris (pH 8.0), 150 mM NaCl, 1% NP40, 5 mM EDTA, 0.5% sodium deoxycholate, 0.1% sodium dodecyl sulfate) and centrifuged at 50'000×g for 20 min at 4°C. The supernatants containing RIPA-soluble proteins were collected and centrifuged again. The pellets from both spins were resuspended in RIPA buffer and centrifuged again. The supernatants were discarded and the pellets were resuspended in 7.5 µl/mg 70% formic acid (FA) in distilled water. Prior to centrifugation at 50'000×g for 20 min at 4°C, samples were incubated for 30 min on ice. The supernatants containing FA-soluble proteins (also considered as RIPA insoluble proteins) were centrifuged again. The FA fractions were dialyzed against PBS over night at 4°C, then an equal volume of 50 mM Tris-HCl, pH 7.4 was added to each sample. Protein concentrations were determined using a DC Protein Assay kit (BioRad, USA).

Furthermore, we extracted insoluble proteins from brains with sarkosyl, following a method described previously [26], [37]. Briefly, hemispheres were weighed and homogenized in 6 µl/mg salt buffer (100 mM MES (pH 6.8), 750 mM NaCl, 1 mM EGTA, 0.5 mM MgSO_4_, 1 mM dithriothreitol and protease inhibitors). Homogenates were incubated for 20 min at 4°C and then centrifuged at 11'000×g for 20 min at 4°C. The supernatant was then centrifuged at 100'000×g for 60 min at 4°C. The supernatant contained soluble proteins. For the isolation of insoluble proteins, the pellets were twice extracted in 1∶10 (w/v) extraction buffer (10 mM Tris (pH 7.4), 10% sucrose, 850 mM NaCl, 1 mM EGTA) and centrifuged at 15'000×g for 20 min at 4°C. The supernatants were combined and sarkosyl was added to a final concentration of 1%, followed by 1 hour of incubation at room temperature on a head-over-head rotor. The samples were then centrifuged at 100'000×g for 45 min at 4°C to pellet the sarkosyl-insoluble proteins, which were resuspended in 0.4 µl/mg 50 mM Tris-HCl, pH 7.4. All chemicals were obtained from Sigma (USA), unless otherwise stated.

### Western Blotting

Western blotting was carried out as previously described [38]. Briefly, proteins were separated by SDS-PAGE (Tetracell, BioRad, USA) and electro-transferred onto nitrocellulose membranes (Amersham, USA). The membranes were blocked with 5% bovine serum albumin (BSA) in 50 mM Tris, pH 7.4, containing 0.1% Tween-20 for 1 hour at room temperature, before incubation with primary antibodies in blocking buffer over night at 4°C. Primary antibodies were against tau (Table 1), GAPDH (Chemicon, USA) and actin (Sigma, USA). Bands were visualized, using alkaline phosphatase-coupled secondary antibodies, together with the Immobilon^TM^ Western substrate (Millipore, USA) in a VersaDoc 4000 imaging system (BioRad, USA). Bands were quantified with the Quantity One 1-D software v4.6 (BioRad, USA).

### Histology

Immunohistochemistry was carried out as previously described [39], [40]. Briefly, 4% paraformaldehyde (Sigma, USA) fixed tissue from transcardially PBS perfused mice were embedded in paraffin using an Excelsior tissue processor (Thermo, USA) and sectioned at 5 µm. For standardization, all stainings were carried out in Sequenza racks (Thermo, USA). Prior to blocking with PBS containing 3% heat inactivated goat serum and 5% BSA for 1 hour at room temperature, antigen retrieval was performed in 10 mM citrate buffer, pH 5.8 in a RHS-1 microwave vacuum histoprocessor (Milestone, USA) at 120°C. Sections were incubated over night at 4°C with primary antibodies in blocking buffer. Primary antibodies were against tau targeting a range of epitopes (Table 1), insulin and glucagon (both Sigma, USA). Antibody staining was visualized using Alexa 555- and 488-coupled secondary antibodies (Invitrogen, USA), together with nuclear marking by 4′,6-diamidino-2-phenylindole (DAPI, Invitrogen, USA). Pictures were taken with a BX51 fluorescence microscope equipped with a DP70 CCD color camera (Olympus, USA). Gallyas silver staining to reveal NFTs was performed following a standard protocol [27]. Hematoxylin/eosin staining was done following standard protocol. For quantification of NFTs and immunofluorescence, serial sections were stained and analyzed as previously described [22].
